# Phorbol Ester Modulation of Ca^2+^ Channels Mediates Nociceptive Transmission in Dorsal Horn Neurones

**DOI:** 10.3390/ph6060777

**Published:** 2013-05-29

**Authors:** Li Yang, Iqbal Topia, Toni Schneider, Gary J. Stephens

**Affiliations:** 1 School of Pharmacy, University of Reading, Whiteknights, Reading, PO Box 228, RG6 6AJ, UK; E-Mails: yanglidyo@hotmail.com (L.Y.); iqbal.topia@gmail.com (I.T.); 2 School of Basic Medical Science, Liaoning University of Traditional Chinese Medicine, Shenyang 110847, China; 3 Institute of Neurophysiology, University of Cologne, Robert-Koch-Strasse 39, Köln D-50931, Germany; E-Mail: akp14@uni-koeln.de

**Keywords:** phorbol ester, protein kinase C, Ca^2+^ channel, nociception, patch-clamp

## Abstract

Phorbol esters are analogues of diacylglycerol which activate C1 domain proteins, such as protein kinase C (PKC). Phorbol ester/PKC pathways have been proposed as potential therapeutic targets for chronic pain states, potentially by phosphorylating proteins involved in nociception, such as voltage-dependent Ca^2+^ channels (VDCCs). In this brief report, we investigate the potential involvement of Ca_V_2 VDCC subtypes in functional effects of the phorbol ester, phorbol 12-myristate 13-acetate (PMA) on nociceptive transmission in the spinal cord. Effects of PMA and of selective pharmacological blockers of Ca_V_2 VDCC subtypes on nociceptive transmission at laminae II dorsal horn neurones were examined in mouse spinal cord slices. Experiments were extended to Ca_V_2.3(−/−) mice to complement pharmacological studies. PMA increased the mean frequency of spontaneous postsynaptic currents (sPSCs) in dorsal horn neurones, without an effect on event amplitude or half-width. sPSC frequency was reduced by selective VDCC blockers, ω-agatoxin-IVA (AgTX; Ca_V_2.1), ω-conotoxin-GVIA (CTX; Ca_V_2.2) or SNX-482 (Ca_V_2.3). PMA effects were attenuated in the presence of each VDCC blocker and, also, in Ca_V_2.3(−/−) mice. These initial data demonstrate that PMA increases nociceptive transmission at dorsal horn neurones via actions on different Ca_V_2 subtypes suggesting potential anti-nociceptive targets in this system.

## 1. Introduction

Chronic pain states continue to represent debilitating illnesses with a significant unmet clinical need. Amongst potential new therapeutic leads, agents that activate PKC have been proposed as anti-nociceptive agents [1]; in this regard, phorbol esters, which are a class of non-hydrolysable analogues of diacylglycerol, activate PKC experimentally. In a related manner, targeting presynaptic VDCCs in nociceptive pathways is another potentially beneficial therapeutic avenue [2,3]. VDCCs may be divided into three major families, Ca_V_1-3; the Ca_V_2 family (Ca_V_2.1, Ca_V_2.2 and Ca_V_2.3) are predominantly localised to presynaptic nerve terminals, where they control transmitter release and different aspects of synaptic plasticity [4,5,6]. Within the dorsal horn of the mammalian spinal cord, Ca_V_2.1 and Ca_V_2.2 subunits are strongly expressed in presynaptic terminals, whilst there is also evidence for presynaptic expression of Ca_V_2.3 subunits in nociceptive pathways [7,8,9]. The phorbol ester/PKC pathway has been proposed to phosphorylate Ca_V_2 subunits in expression systems [10,11,12,13]. The molecular determinants for the phorbol ester/PKC pathway include sites on the intracellular linker between domains I and II (the I-II loop) of Ca_V_2 subunits, this pathway is involved in regulating transmitter release, potentially by opposing inhibitory G protein modulation, as reviewed in [14,15]. This dynamic regulation may be therapeutically targeted in nociceptive pathways. However, phorbol esters may also activate other “non-kinase” C1 domain-containing receptors [16]; therefore, a major aim of this study was to determine if a phorbol ester pathway involving Ca_V_2 VDCCs operates in nociceptive pathways.

We have previously shown that isolated dorsal root ganglia (DRG) neurones associated with the Aδ and C fibres that supply laminae I and II in the dorsal horn contain Ca_V_2.1, Ca_V_2.2 and Ca_V_2.3 current components (as defined by sensitivity to selective pharmacological agents) [17]. Moreover, *in vivo* electrophysiological recording from wide-dynamic range neurones located in rat lamina V dorsal horn suggest that Ca_V_2.2, and to a lesser extent Ca_V_2.1, channels contribute to basal synaptic transmission, and that contributions of Ca_V_2.2 are increased under neuropathic conditions [18]. Ca_V_2.3 plays only a minor role to basal transmission at WDR neurones, but neuropathy also causes a clear increase in Ca_V_2.3 contribution to nociceptive transmission [19]. Here, we demonstrate that Ca_V_2.1, Ca_V_2.2 and Ca_V_2.3 subunits represent molecular targets in phorbol ester-stimulated increases in nociceptive transmission onto lamina II dorsal horn neurones; such findings are consistent with a pathway involving phorbol ester/PKC modulation of VDCCs in the mammalian spinal cord.

## 2. Experimental Section

### 2.1. Acute spinal Cord Slice Preparation and Solutions

Spinal cord slices were prepared from three to four week-old male litter-matched C57Bl/6 wild type or Ca_V_2.3(−/−) mice [20]. Mice were anesthetized by isofluorane inhalation and humanely killed by cervical dislocation and decapitated, in line with UK Home Office procedures [Animals (Scientific Procedures) Act 1986] and as approved by Local Ethical Review Panel. A lumbar laminectomy was performed and the spinal cord was gentled excised under pressure from a syringe filled with ice-cold sucrose-based artificial cerebrospinal fluid (aCSF) solution containing (in mM): sucrose 124, KCl 3, NaHCO_3_ 26, NaH_2_PO_4_ 2.5, MgSO_4_ 2, CaCl_2_ 2, D-glucose 10 (pH 7.3). The spinal cord was placed in a shallow-grooved agarose block which was glued to the stage of a Vibroslice 725M (Campden Instruments Ltd, Loughborough, UK) and transverse slices (300 μM) cut in ice-cold sucrose-based aCSF bubbled with 95% O_2_/5% CO_2_. Slices were transferred into standard aCSF solution contained (in mM): NaCl 124, KCl 3, NaHCO_3_ 26, NaH_2_PO_4_ 2.5, MgSO_4_ 2, CaCl_2_ 2, D-glucose 10, maintained at pH 7.3 by bubbling with 95% O^2^ / 5% CO_2_ at 37 °C for 1 h and then maintained at room temperature (20–24 °C).

### 2.2. Electrophysiological Recording

Spinal cord slices were transferred to a recording chamber on an upright Olympus BX50WI microscope (Olympus, Tokyo, Japan) and perfused at 2–4 mL min^−1^ with standard carboxygenated aCSF at room temperature. The high neuronal density allowed identification of laminae II, where individual dorsal horn neurones were visualised using differential interference contrast optics with a 60x water immersion lens. Whole-cell patch clamp recordings were performed using an EPC-10 patch-clamp amplifier (HEKA Electronik, Lambrecht, Germany), controlled by PatchMaster software (HEKA). Patch electrodes were fabricated from borosilicate glass (GC150-F10, Harvard Apparatus, Kent, UK) using a Flaming Brown P-87 micropipette puller (Sutter Instruments Company, Novato, CA, USA) and fire-polished using a MF-830 Microforge (Narashige, Tokyo, Japan). Electrodes had resistances 7-8 MΩ when filled with an intracellular solution [containing (in mM): CsCl 140, MgCl_2_ 1, CaCl_2_ 1, EGTA 10, MgATP 4, NaGTP 0.4, HEPES 10, pH 7.3 with CsOH]. Series resistance was typically less than 20 MΩ and compensated by 70%–90% throughout. Data were sampled at 7 kHz and filtered at one-third of the sampling frequency. In this study, rapidly activating, inward currents at a holding potential of −70 mV were designated as sPSCs. In the present brief report we did not attempt to distinguish between effects on inhibitory glycinergic or GABAergic or excitatory glutamatergic transmission. Data were analysed using FitMaster (HEKA), Axograph (Molecular Devices, Sunnyvale, CA, USA) and Excel (Microsoft, Redmond, WA, USA) software. Cumulative frequency plots were constructed for sPSC inter event intervals using 5 ms bins. sPSC frequency data were normalised to control period (0–5 mins prior to drug treatment) and measured at steady-state (10–15 mins after drug treatment). Effects on sPSC amplitude (as a measure of effects on postsynaptic receptor sensitivity) and on sPSC half-width (as a measure of effects on clearance of transmitter from the synaptic cleft *i.e.*, a reflection of any change in timecourse of event decay) were also determined. Data are presented as mean value ± S.E.M. Statistical significance was determined using Student's paired or unpaired *t*-tests, Kolmogorov-Smirnov (KS) tests or Mann-Whitney U-tests, as described in text. In all cases, *p* < 0.05 was considered significant.

### 2.3. Pharmacology

The following agents were purchased: PMA (Sigma, Poole, UK); ω-conotoxin GVIA (Alomone Labs, Jerusalem, Israel); ω-agatoxin IVA (Sigma); SNX-482 was a kind gift from Alomone Labs. All drugs were made up at 1000 x stocks in distilled water, except PMA, which was dissolved in DMSO. Stocks were aliquoted and stored at −20 °C. Aliquots were thawed and dissolved in carboxygenated aCSF immediately prior to use.

## 3. Results and Discussion

### 3.1. Effect of PMA on Synaptic Transmission in Dorsal Horn Neurones

**Figure 1:**
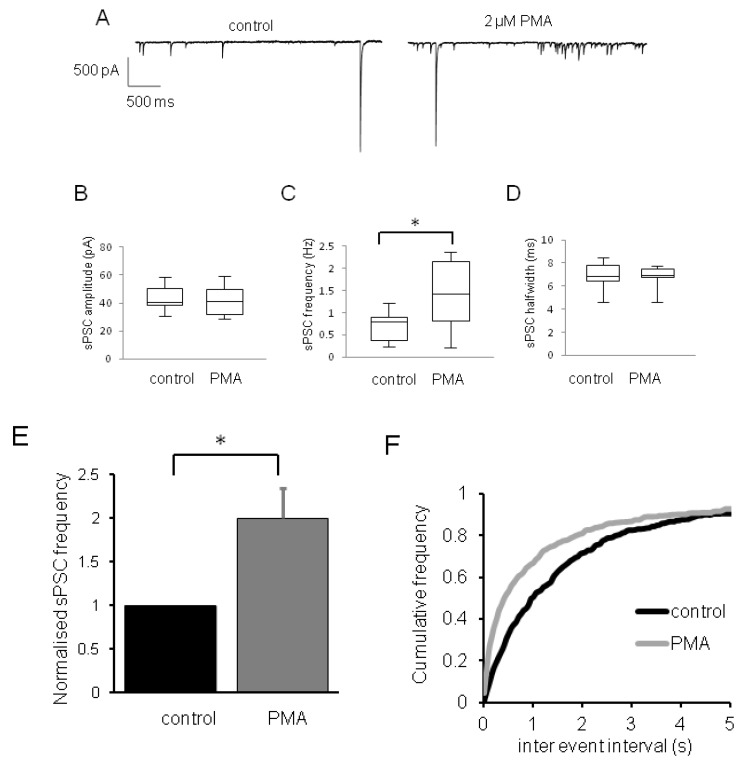
Effects of PMA on nociceptive transmission in dorsal horn neurones. (**A**) Raw data traces showing effects of PMA (2 M) on control sPSCs (holding potential (V_H_) = −70 mV). Box and whisker plots showing effects of PMA (2 μM) on (**B**) sPSC amplitude (**C**) sPSC frequency and (**D**) sPSC half-width. In these plots, the median value is represented by central bars, 25% and 75% quartile ranges depicted by boxes, max/min values represented by “error” bars. (**E**) Bar graph showing summarised effects of PMA (2 μM) on normalised sPSC frequency. (**F**) Cumulative frequency plots for effect of PMA (2 μM) on control sPSC inter event interval; data sets significantly different using *KS*-test. (B–F) *n* = 6 ± S.E.M., * = *p* < 0.05 (Mann Whitney *U*-test).

Whole-cell recordings were made from visually identified laminae II dorsal horn neurones within the substantia gelatinosa in spinal cord slices. Bath application of the phobol ester PMA (2 µM) caused a significant increase in mean sPSC frequency (Figure 1A,C,E,F, Table 1), but was without effect on mean sPSC amplitude (Figure 1B, Table 1) and half-width (Figure 1D, Table 1). Phorbol esters such as PMA are widely used to activate PKC isoforms, a family of at least 10 serine/threonine kinases that have been proposed as major regulators of sensitization in chronic pain states [1]. For example, phorbol ester-activated PKC isoforms are known to be up-regulated in dorsal horn neurones in response to noxious stimuli [21]. Treatment with phorbol esters has been widely used to implicate PKC isoforms as phosphorylating agents at VDCCs, such actions potentiate Ca^2+^ current [10,22], consistent with phorbol ester-induced increases in vesicular release seen here. However, PMA/PKC phosphorylation pathways also have potential to target other ion proteins at nociceptive presynaptic terminals, including AMPA, NMDA and kainate ionotropic receptors, transient receptor potential channel subtypes [23,24,25] and, also, other C1 domain-containing receptors potentially involved in exocytosis [26,27]. Such studies suggest that phorbol ester-mediated effects on synaptic transmission may occur, at least in part, independently of effects on VDCCs; therefore, a major aim of the present study was to investigate whether (i) presynaptic Ca_V_2 VDCCs mediate PMA effects in nociceptive pathways and (ii), if so, which Ca_V_2 subtypes are predominantly involved.

**Table 1:** Effects of PMA on sPSCs in dorsal horn neurones in litter-matched WT and Ca_V_2.3(−/−) mice.

	sPSC amplitude (pA)	sPSC frequency (Hz)	sPSC half-width (ms)
WT (n = 6)	43.5 ± 4.2	0.69 ± 0.16	6.85 ± 0.57
PMA (n = 6)	41.8 ± 5.0	1.40 ± 0.36 *	6.72 ± 0.47
Ca_V_2.3(−/−) (n = 8)	57.3 ± 9.7	1.00 ± 0.15	7.26 ± 0.42
PMA (n = 8)	47.4 ± 5.3	1.08 ± 0.21	7.27 ± 0.40

No significant differences were seen in basal sPSC properties between dorsal horn neurones from WT and Ca_V_2.3(−/−) mice (Student's unpaired *t*-tests). PMA caused a significant increase in sPSC frequency in dorsal horn neurones from WT, but not Ca_V_2.3(−/−) mice. *= *p* < 0.05 *vs* control (WT) (Student’s paired *t*-test).

### 3.2. Contribution of Ca_V_2 VDCC Subtypes to PMA Effects on Synaptic Transmission in Dorsal Horn Neurones

**Figure 2:**
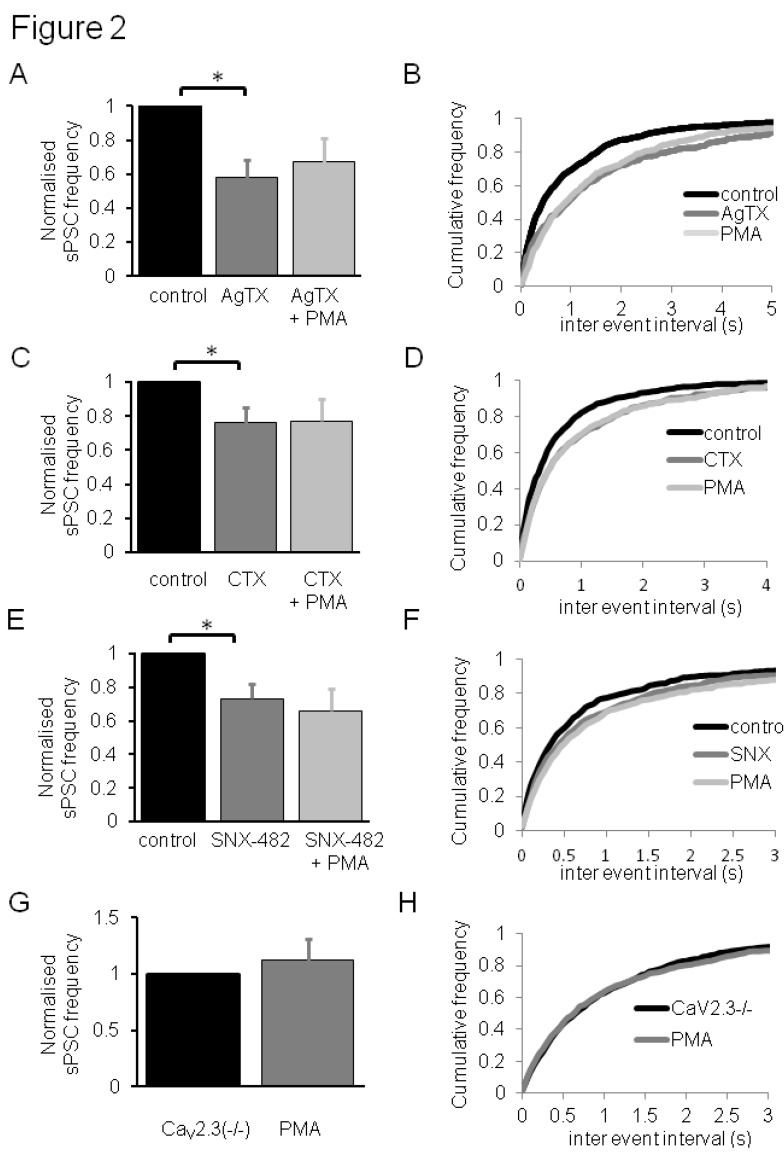
Contribution of Ca_V_2 VDCC subtypes to PMA effects on nociceptive transmission in dorsal horn neurones. Bar graphs showing summarised effects of selective VDCC blockers effects on normalised sPSC frequency and PMA (2 µM) actions in the presence of (**A**) 200 nM AgTx (n = 6 ± S.E.M.), (**C**) 1 µM CTX (n = 6 ± S.E.M.) and (**E**) 300 nM SNX-482 (n = 6 ± S.E.M.). (**G**) Bar graph showing summarised effects of PMA (2 μM) effects on normalised sPSC frequency in Ca_V_2.3(−/−) mice (n = 8 ± S.E.M.). In all cases, * = *p* < 0.05 (Mann Whitney *U*-test). Cumulative frequency plots showing summarised effects of selective VDCC blockers effects on sPSC inter event interval and PMA (2 μM) actions in the presence of (**B**) 200 nM AgTx (n = 6 ± S.E.M.), (**D**) 1 μM CTX (n = 6 ± S.E.M.) and (**F**) 300 nM SNX-482 (n = 6 ± S.E.M.); each blocker caused a significant decrease in inter event interval, PMA had no further effect in each case using *KS*-tests. (H) Cumulative frequency plot showing summarised effects of PMA (2 μM) effects on normalised sPSC frequency in Ca_V_2.3(−/−) mice (n = 8 ± S.E.M.); data sets not significantly different using *KS*-test.

Bath application of 200 nM AgTX (Figure 2A,B), 1 μM CTX; (Figure 2C,D) or 300 nM SNX-482 (Figure 2E,F) each caused a significant inhibition in mean sPSC frequency; none of these selective VDCC blockers had significant effects on mean sPSC amplitude or half-width (data not shown). Initially, these data are consistent with high-voltage-activated Ca_V_2.1, Ca_V_2.2 and Ca_V_2.3 VDCC subtypes each contributing to nociceptive transmission in lamina II dorsal horn neurones. In line with such observations, intrathecal injection of AgTX, CTX or SNX-482 has been shown to have anti-nociceptive effects *in vivo*, also consistent with immunohistochemistry localization of Ca_V_2 family subunits to the dorsal horn of the spinal cord [8]. Presynaptic Ca_V_2.2 (N-type) Ca^2+^ channels have been shown to make a major contribute to nociception [18,28] and mediate excitatory glutamate release onto laminae I/II dorsal horn neurones [29,30,31] and play a major role in opioid-mediated inhibition [32]; in these studies, Ca_V_2.1 (P/Q-type current) subunits also play, an albeit more minor, role. A CTX- and AgTx-resistant (R-type) Ca^2+^ current component in dorsal horn neurones, potentially due to Ca_V_2.3 subunits, has also been reported [31]. We have previously shown that Ca_V_2.3 subunits contribute to R-type Ca^2+^ current in DRGs cells and that Ca^2+^ currents encoded by Ca_V_2.3 subunits are blocked by SNX-482 [17]. Although much less is known about the contribution of Ca_V_ subtypes to inhibitory transmission, Rycroft and co-workers [31] reported that N-type currents contribute to evoked IPSCs in lamina I neurones, however, unlike for excitatory inputs, this contribution was unchanged under neuropathic conditions. Finally, it is worth pointing out that low-voltage-activated, T-type Ca_V_3.2 channels have also been shown to contribute to nociceptive transmission in the dorsal horn neurones [33].

Bath application of 2 μM PMA in the continued presence of either 200 nM AgTX (Figure 2A,B), 1 μM CTX; (Figure 2C,D) or 300 nM SNX-482 (Figure 2E,F) had no effect on mean sPSC frequency; under these conditions, 2 μM PMA also had no effect on mean sPSC amplitude or half-width (data not shown). These data implicate different Ca_V_2 VDCCs as mediators of PMA effects on nociceptive transmission in the dorsal horn. It is of interest that blockade of an individual Ca_V_2 subtype was sufficient to removal the stimulatory effect of PMA; this sensitivity suggests that, in terms of PKC response, there is a lack of Ca_V_2 subtype redundancy within this system. Phorbol esters have been used to implicate serine and threonine residues in the VDCC I-II loop and C-terminal as important determinants for PKC-mediated potentiation of Ca^2+^ current in Ca_V_2.2 and Ca_V_2.3 channels [10,11,12,13]. Ca_V_2.1 subunits were reported to be unaffected by PMA [10,22]; however, this effect may be dependent on the PKC isoform activated, the availability of isoform-dependent phosphorylation sites or even the channel environment, as these experiments were performed in expression systems. In regard to the latter, there is evidence that AgTx attenuates PMA-mediated increases in transmitter release at the neuromuscular junction [34,35] and we have previously shown that phorbol esters cause a significant increase in inhibitory synaptic transmission at the interneurone-Purkinje cells pathway in the cerebellum [36], synapses at which Ca_V_2.1 channels dominate the control of transmitter release [37]. The functional data presented here suggest that PMA can exert effects on endogenous AgTx-sensitive Ca_V_2.1 channels in nociceptive pathways.

Original studies using cloned, isolated ion channels in expression systems by Newcomb and co-workers [38], have prompted researchers to use 300 nM SNX-482 as a selective concentration for Ca_V_2.3 over other channels, including Cav2.1; however, Arroyo *et al.* [39] have suggested that 300 nM SNX 482 may also affect Cav2.1; therefore, the effects of PMA were also examined in Ca_V_2.3(−/−) mice. In this regard, Ca_V_2.3(−/−) mice were shown to have selective alterations in behavioural pain responses [40]. 2 μM PMA was without effect on mean sPSC frequency (Figure 2G,H), amplitude or half-width (Table 1) in Ca_V_2.3(−/−) mice. These genetic deletion data support pharmacological data implicating a role for Ca_V_2.3 subunits in PMA actions. In our experiments, genetic deletion of Ca_V_2.3 *per se* had no clear effect on mean sPSC frequency, amplitude or half-width in comparison to litter-matched wild-type mice used in the experiments above (Table 1). This may be unexpected in relation to the reduction in sPSC frequency seen for SNX-482 above and we cannot rule out a compensatory upregulation of other Ca_V_2 isoforms; alternatively, we previously reported that small proportion of DRG Ca^2+^ current in mice lacking Ca_V_2.3 is sensitive to SNX-482 [17], so it possible that SNX-482 may also be blocking further VDCC targets here.

## 4. Conclusions

The major conclusions of this brief report are that different Ca_V_2 VDCC subtypes contribute to nociceptive transmission in lamina II dorsal horn neurones and that these Ca_V_2 channels represent molecular targets for the stimulatory effects of PMA on nociceptive transmission; we also indicate a lack of Ca_V_2 subtype redundancy in terms of PMA responses. A potential mechanism whereby PMA exerts effects is via the activation of PKC isoforms, leading to phosphorylation of presynaptic Ca_V_2 subunit in dorsal horn neurones to modulate excitability. Here, we record mixed sPSC responses to determine overall PMA effects on nociceptive transmission; in the future, it will be important to determine PMA actions on different inhibitory glycinergic or GABAergic and/or excitatory glutamatergic pathways and, also, to determine if other Ca_V_ channels are a further target for PMA effects on nociceptive transmission in the continuing search for novel therapeutic targets in pain.
